# Transcriptome Analysis of Sugarcane Response to Sugarcane Yellow Leaf Virus Infection Transmitted by the Vector *Melanaphis sacchari*

**DOI:** 10.3389/fpls.2022.921674

**Published:** 2022-06-14

**Authors:** Rubab Shabbir, Lin Zhaoli, Xu Yueyu, Sun Zihao, Chen Pinghua

**Affiliations:** ^1^Key Laboratory of Sugarcane Biology and Genetic Breeding, Ministry of Agriculture, National Engineering Research Center of Sugarcane, Fujian Agriculture and Forestry University, Fuzhou, China; ^2^Key Laboratory of Ministry of Education for Genetics, Breeding and Multiple Utilization of Crops, College of Agriculture, Fujian Agriculture and Forestry University, Fuzhou, China

**Keywords:** transcriptome analysis, sugarcane, sustainability, aphid vector, ScYLV

## Abstract

Sugarcane yellow leaf disease severely affects sugarcane production. As a viral disease, the pathogen sugarcane yellow leaf virus can only be transmitted by aphid vectors rather than mechanical means. To understand the sugarcane responses to ScYLV infection, the corresponding transcriptomic profile of ScYLV-infected and ScYLV-free plants were analyzed with RNA-Seq technology. In this study, *Melanaphis sacchari* was used as the vector to transmit ScYLV to the susceptible sugarcane cultivar CP72-1210 and transcriptome was sequenced as well as differentially expressed genes between disease-infected and non-infected sugarcane plants were investigated. A total of 1,22,593 genes were assembled, of which 1,630 genes were differentially expressed. Among DEGs, 1,622 were upregulated and eight were downregulated that were further annotated with GO, KEGG, KOG, PFAM, SwissProt, and Nr databases. The expression levels of DEGs in the three KEGG pathways, namely endocytosis, PEX protein synthesis, and endoplasmic reticulum stress response to viral protein synthesis were observed. Interestingly, it was found that the yellow leaf virus could induce the formation of autophagosomes by LC3, promoted by ER stress, and may be related to the replication of viral RNA. We tested 63 DEGs in this research. The qRT-PCR results showed that two were downregulated and 45 were upregulated in response to the ScYLV infection. This study will not only offer an overall comprehension of sugarcane responses to ScYLV infection at the gene expression level but also increase the chances to block the transmission of ScYLV for use in further molecular biology techniques and will aid in increasing the resistance of plants against ScYLV.

## Introduction

Sugarcane yellow leaf disease (ScYLD) also known as sugarcane yellow leaf syndrome (ScYLS), has an incidence ranging from 10 to 96% in sugarcane growing countries worldwide. The disease is also quite common and intense in sugarcane planting areas of China resulting in a 40–60% loss in sugarcane production. CP varieties are the main cross parents in sugarcane breeding programs in China, but the CP series are highly susceptible to yellow leaf disease and their progeny has also shown susceptibility toward this disease. Overall, the symptoms of this disease include intense yellowing of the lower side of the sugarcane leaf midrib and necrosis proceeding from the tip toward the base of the leaf (Bertani et al., 2014). The typical symptom of diseased leaves is the change from green to yellow coloration from the lower part of the midrib. The upper surface of the midrib remains normal white or white with green, pink, or reddish coloration. However, the pink color of the midrib is not constant. The midrib eventually turns yellow when the new leaf grows. In addition, the symptoms of the disease vary depending on the genetic background of the species. The symptoms of ScYLD first reported in the Hawaiian Islands involved yellowing of the midribs and necrosis of leaf tips that continue to spread throughout the leaf blade until the entire leaf appears yellow or even necrotic (Grisham et al., 2010). Red spots on the midrib of some plant leaves have also been observed. The entire leaves of some species have also appeared yellow after infection with yellow leaf syndrome. Yellowing spreads from midrib to both sides of the leaf blade in some varieties growing in the fall and winter seasons. The degree of yellowing also increases with the change in temperature and time (ElSayed et al., 2010).

Sugarcane yellow leaf virus (ScYLV) is the causative agent of ScYLD that spreads systematically. The ScYLV granules are icosahedrons with a diameter of 24–29 nm and buoyancy density of 1.30 g/m^3^. It has ssRNA composed of a protein that wrapped single-stranded RNA (Madugula and Gali, 2017). This research indicates that six open reading frames, namely ORF0 to ORF5, have a significant impact on the virus infecting a host. The ORF0 locates at the 5′ end of the genome and encodes the P0 protein. Research on the P0 protein has focused on exploring its function as a silencing suppressor. The F-box domain in the P0 protein is the target protein that inhibits the gene silencing of the active site F-box domain. The target protein is the plant AGO1 which causes the components of the silencing complex to fail to assemble appropriately and eventually. The AGO1 protein cannot function and is degraded by the ubiquitin degradation system of plants. The ORF3 encodes a capsid protein CP or P3, while ORF5 encodes a read-through protein and the genomic RNA of the YLV is coated with these two structural proteins to form an icosahedron with a diameter of 22–23 nm regular sphere observed under an electron microscope (ElSayed and Komor, 2013). On the surface of intact virions, most of them are CP and a few are CP-RTP. Viruses of the *Luteoviridae* family complete the long/short-distance movement in the phloem of the plant and the transmembrane transport in the larvae in the form of intact virions. The process of virus particle assembly and initial infestation of plants does not require CP-RTP, but CP-RTP only binds to synthetic virions that belong to the *cis* group. When the virus infects the plant, the N-terminal region of RTP participates in the systematic transport of the virus, the accumulation of the C-terminal region, the number of virus particles, and restricts the activity range of the virus particles to be only in the vascular bundles. Although the roles of various regions of RTP are obvious, the mutation experiments of the three viruses of the family *Luteoviridae*, namely BWYV, BYDVPAV, and PLRV have shown that only complete CP-RTP can ensure the morphological structure of the virus particles to be stable, thus ensuring its normal accumulation in the plants and the spread to uninfected areas have long been studied. It has further been shown that the CP-RTP binding protein of the YLV plays an important role not only in the transportation within plants but also in the body of aphid mediators (Fartek et al., 2014).

**FIGURE 1:**
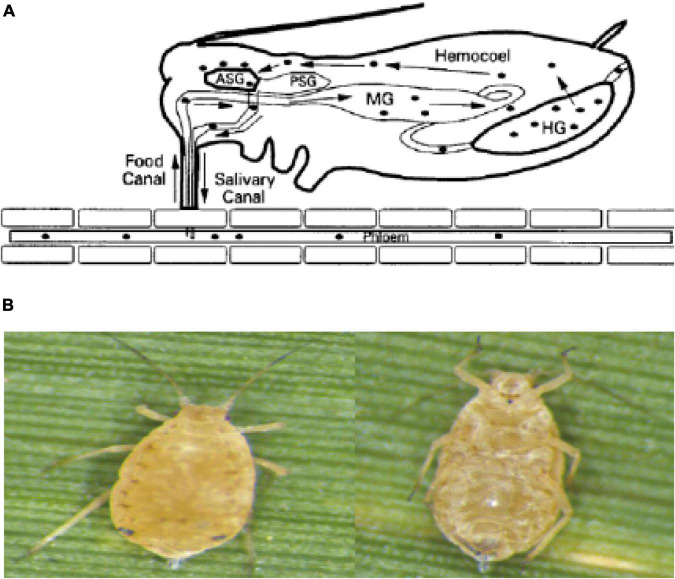
**(A)** The path of the virus in aphids. **(B)** The aphid’s vector of ScYLV. Left: back view of the aphid. Right: abdomen view of the aphid.

Research showed that ScYLV is transmitted without mechanical friction and can be transmitted persistently by *Melanaphis sacchari* and *Ceratovacuna lanigera*. *Siha flavor* and *Schizaphis graminum* cannot be infected under natural conditions. ScYLV only exists in some species of the sugarcane genus but under artificial inoculation conditions, it can successfully transmit from sorghum to some strains of wheat, oats, barley, rice, corn, and sugarcane that are closely related. *R. rufiabdominalis* can transmit the virus among wheat plants (Zhu et al., 2011). Vector or insect-borne plant viruses can be classified as non-persistent, persistent, and semi-persistent viruses based on the length of the virus that the mediator insects use to spread the virus. Among them, persistent viruses can be divided into persistent non-proliferative viruses and persistent proliferative viruses (Brault et al., 2010). After the vector insects feed on the virus-infected host plants, the virus particles enter the insect esophagus from the mouth with the plant sap, pass through the foregut to the mid-gut, and combine with the carrier to penetrate the epithelial cells of the mid-gut or hindgut into the hemolymph. They enter in the salivary glands of the vector through hemolymph membrane. When the insects feed again, the virus is released with saliva to the new host phloem tissue to complete effective transmission as shown in Figure 1A (ElSayed et al., 2016).

Studies have shown that there are 621 species of plant viruses known worldwide, but only 272 species are known to be vectored by the insects. Most of the insect vectors are concentrated in the family Homoptera, most of which are aphids. So, the most common types of viruses are transmitted through the aphids. Ticks are also important as mediators of plant pathogenic viruses (Giordanengo et al., 2010). Some aphid species can spread several hundreds of viruses. For example, *Myzus persicae* can transmit 115 different kinds of viruses while *M. sacchari* can spread 58 viruses and cause plant diseases. Different kinds of aphids have different efficiency levels for the transmission of the YLV (Pirone and Harris, 1977). In this regard, *M. sacchari* has the highest transmission efficiency as a vector to transmit non-persistent viruses. Different sugarcane plants also have different resistance to YLV. For instance, CP species from the United States are highly susceptible to YLV. However, ROC-24 is highly resistant to the yellow leaf virus (Zhou and Li, 2012). So, why are only aphids able to transmit ScYLV, rather than other ways, like mechanical means? What genes are involved in the recognition of sugarcane plants to ScYLV? Therefore, in this research study, a susceptible sugarcane variety CP72-1210 was infected by ScYLV with the help of aphids and the total RNAs were sequenced to understand the response of sugarcane plants to ScYLV infection at the gene expression level. Moreover, some genes playing an essential role in the virus infection process will be identified to create transgenic plants resistant to SCYLV infection and to control the further spread of the disease.

## Materials and Methods

### Aphid Collection and Inoculation With ScYLV

*Melanaphis sacchari* (Z) was obtained from the National Engineering Research Center of Sugarcane, Fujian Agriculture and Forestry University, Fuzhou, China (26.0849°N, 119.2397°E). The sugarcane leaves with a single colony of *M. sacchari* were cut off and kept in glass bottles with pure water at the bottom, then maintained at 20°C and 70% humidity and a 16 h light: 8 h dark period in an artificial climate incubator (Ningbo Haishu Saifu Experimental Instrument Factory, PRX-450A, China). The collected aphids were oval (Figure 1B), light yellow to black, the antennae were slender with 6 knots and the compound eyes were large and brownish-red. In the central part of the abdomen, 3–6 internodes had large rectangular spots, and the abdominal tube was brown and cylindrical. The tailpiece was conical, blunt, and slightly thicker in the middle (Salokhe, 2021). Some of the colonies were fed with leaves of ScYLV-free CP72-1210 which were considered controls, and some were fed with ScYLV-infected leaves. The successful infection of aphids with ScYLV was confirmed by PCR.

### Heat Treatment of CP72-1210 for Clean Cane

The sugarcane variety CP72-1210 was obtained from the National Engineering Research Center of Sugarcane (Fujian Agriculture and Forestry University) and was selected as a host for artificial inoculation because of its susceptibility to the ScYLV. The variety is also one of the main crossing parents in sugarcane breeding programs in China. According to the relevant literature, heat treatment is an excellent method to get virus-free seedlings since sugarcane cultivars are usually infected by pathogens. The detoxification treatment of stalks at 52°C for 30 min proved to be a better treatment to ensure the average germination rate of the stems above 60% and no yellow leaf virus was detected at the growth point of sugarcane buds (ElSayed et al., 2014). One complete stem of CP72-1210 was cut into short stalks with two nodes and heat-treated following the protocol described above and then placed in an incubator at 38°C for 1 week germination. The germinated seedlings were then planted in pots with soil (PINDSTRUP SUBSTRATE, 06120806) in a greenhouse maintained at 24°C and 75% humidity and a 16 h light: 8 h dark period. Half of the germinated seedlings from the same single stem at the four-leaf stage were inoculated with 15–20 ScYLV-infected aphids for 3 days. Meanwhile, the rest half of the seedlings were inoculated with the same number of ScYLV-free aphids. After 2 weeks, all the seedlings were detected through RT-PCR to check the presence of ScYLV.

### RNA Extraction and Reverse Transcription

Total RNAs were extracted from all the infected and non-infected seedlings and aphids using the Plant RNA Kit R6827 (OMEGA biotek, China). Meanwhile, all aphids’ samples involved were also subject to total RNA extraction using UNlQ-10 Column Trizol Total RNA Isolation Kit (Sangon Biotech, China) method. Then concentration and purity of all RNA samples were measured on NanoDrop 2000 (ThermoFisher, NanoDrop One, United States) and the integrity of the RNAs was checked on the FUSION FX6 XT (VILBER, VILBER LOURMAT, France). The RNA samples with three bands of 5S, 18S, and 28S were reverse transcribed into cDNA using M-MuLV First Strand cDNA Synthesis Kit (Sangon Biotech, China) and stored at 4°C refrigerators.

### Detection of RNA Quality

The RNA was extracted by the RNA extraction kit and run on an agarose gel electrophoresis for its quality determination which showed three clear and bright bands of equal width with no towing phenomenon. Additionally, the UV spectrophotometer showed a ratio of 260/280 higher than 1.9, a ratio of 260/230 higher than 2.0, and a concentration higher than 500 ng/μl, revealing it is suitable for further reverse transcription.

### Detection of ScYLV in Aphids and CP72-1210

The cDNA samples were tested using the specific PCR primers of YLSF111:5′-TCTCACTTTCACGGTTGACG-3′ and YLSR462:5′-GTCTCCATTCCCTTTGTACACG-3′ to produce a fragment size of 352 bp. PCR master mixture consists of Max Taq DNase 10 μl, 1 μl of primers, 7 μl of pure water, and 2 μl of cDNA. The PCR program was set at 94°C for 2 min followed by 30 cycles of 94°C for the 30 s, 54°C for 30 s, and 72°C for 90 s followed by a final extension for 5 min at 72°C (Hudson, 2008). PCR product amplifications were conducted on PCR System (9700 Applied Biosystems, Inc., Foster City, CA, United States). PCR products were separated by electrophoreses on 1.5% agarose gels containing ethidium bromide (0.5 g/ml) in 0.5 TBE buffer and visualized on a UV. The target fragment was purified, cloned, sequenced, and blast on NCBI.

### Assembly and Annotation of Transcriptomes

Transcriptome sequencing (RNA-Seq) is the use of large-scale sequencing technology to directly sequence cDNA sequences, producing tens of millions of reads, so that the transcription level of a particular genomic region can be directly compared to the genome (Li et al., 2015). Many reads and EST sequences obtained by sequencing in this experiment were processed and spliced to obtain unigenes. By comparing and annotating with public databases, software such as BLAST can be utilized to obtain candidate genes with reference annotation functions or to discover new genes. The DESeq, *p*-value, and Fold Change value were applied to evaluate the biological replicates and to identify DEGs that were significantly different compared to the controls. The data samples were merged and assembled, and contigs were obtained through an overlap of the assembled sequences (Sangon biotech). Then the contigs were clustered according to the paired-end information of sequences and the similarity of contigs. Local assembly was conducted to generate transcripts. The longest transcript in each local region was selected for use as a unigene. For functional annotations, all unigene sequences were first searched against various protein databases such as Nr, SwissProt, COG, and KEGG. To avoid inconsistencies between the unigene alignment results from different databases, the priority was given to SwissProt, KEGG, and COG data sequentially (Mortazavi et al., 2008) setting *q*-value < 0.05, | log2 FoldChange| > 1, further screening DEGs and combining CDD, KOG, COG, NR, NT, PFAM, SwissProt, and TrEMBL databases for gene annotation and biological function annotations and prediction (Mortazavi et al., 2008). The annotated upregulated genes in various metabolic pathways were selected for subsequent functional gene screening based on the KEGG database annotation.

### Screening of Differentially Expressed Genes

According to the RNA-Seq data, differences in mRNA expression levels between control and test plants were identified using the digital gene expression (DGE) profile. Gene expression was quantified as reads per kb per million reads (RPKM). The strict algorithm with *q*-value < 0.05, | log2FoldChange| > 1 was adopted to screen the DEGs. Combined with the functional annotation of DEGs, the KEGG pathway and gene ontology (GO) enrichment analyses as well as the pattern clustering of DEGs, were performed. The results were adjusted using the Bonferroni correction method. Thus, the pathways obtained with obviously enriched DEGs and GO functional categories were further analyzed (Pfaffl, 2001).

### Verification of Differentially Expressed Genes by qRT-PCR

Leaf cDNAs from ScYLV-infected and non-infected plants were used as templates to carry out the qRT-PCR. The specific pairs of primers were designed using the Primer Premier 6.0 software based on qRT-PCR primer design principles and validated for their specificity at NCBI. PCR reaction system was set up according to SYBR Green PCR Master Mix kit (Novogene, Rox, China) instructions using 25SrRNA as an internal reference gene (5′- ATAACCGC-ATCAGGTCTCCAAG -3′; 5′- CCTCAGAGCCAATCCTTTTCC -3′). Each qRT-PCR reaction was performed with 2 μl of cDNA (100 ng/μl), 0.5 μl of each primer, 7 μl water, and 10 μl of PCR master mix in a total volume of 20 μl. The PCR amplifications were done using the following cycling conditions: one cycle at 95°C (5 min), followed by 40 cycles of denaturation at 95°C (10 s), annealing and extension at 60°C for 30 s. Each sample was repeated three times and data were analyzed on ABI 7500 Real-Time PCR System and Software was 2^–Δ^ ^Δ^ *^Ct^* method (Gao et al., 2017).

### Statistical Analysis

The means were compared using the least significance difference (LSD) test at a 5% probability level (*p* ≤ 0.05) with a statistical software package ‘‘Statistix 8.1.’’^1^

## Results

### Virus Detection in Aphids and Plant Samples

**FIGURE 2:**
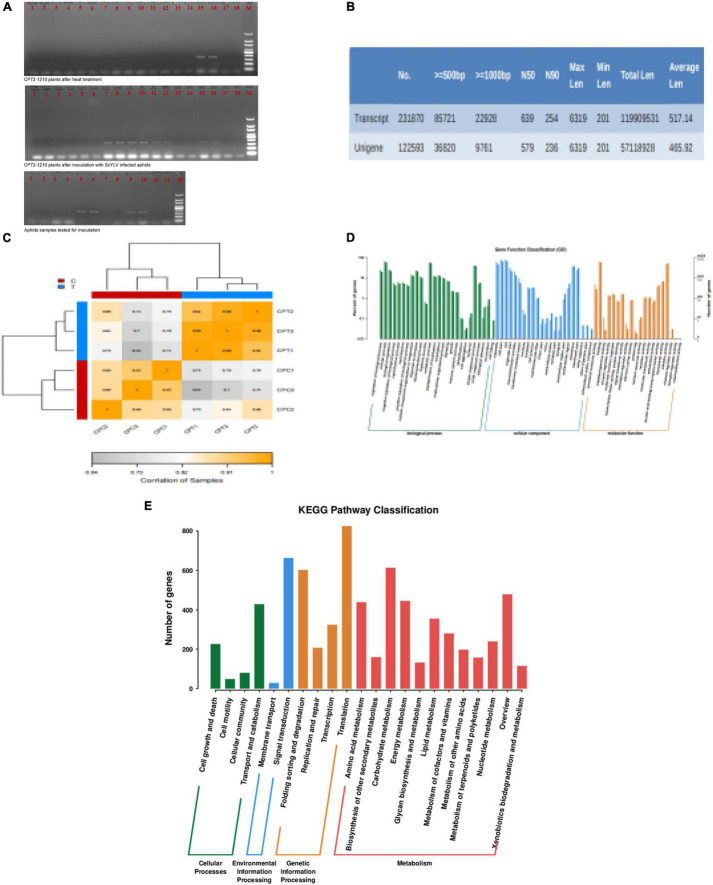
Detection of ScYLV and analysis of RNA-Seq data. **(A)** Ethidium bromide-stained PCR products following amplification with primers YLSF- and YLSR from aphids and CP72-1210 plants. Lane designations: the upper two photos: 1–2: control plant 1; 3–4: control plant 2; 5–6: control plant 3; 7–8: treated Plant 1; 9–10: treated Plant 2; 11–12: treated Plant 3; 13–14: negative check (virus-free plant); 15–16: positive check, and 17–18: blank check (dd H_2_O); M (DNA ladder, Tiangen 100 bp). the photo below: 1–4: aphids fed with leaves of ScYLV-free CP72-1210; 5–6: aphids fed with ScYLV-infected leaves; 7–8: negative check (virus-free plant); 9–10: positive check; 11–12: blank check (dd H_2_O); M (DNA ladder, Tiangen 100 bp). **(B)** Transcript splicing results, including transcript and unigene data number. **(C)** CPT1, CPT2, and CPT3 correlation is all more than 0.94, CPC1, CPC2, and CPC3 also more than 0.89, which proves control and test group correlation meet the experimental requirements. **(D)** Analysis of GO enrichment for genes at the three stages of six samples. The green, blue, and yellow model represents genes number between biological processes, cellular component, and molecular function. **(E)** KEGG pathway enrichment analysis of significant genes. The red, yellow, green, and blue model represents metabolism, genetic information processing, environmental information processing, and cellular processes.

The cDNA samples from both infected aphids and CP72-1210 plants along with their checks were tested by PCR using specific pairs of primers. The PCR amplification products were separated by electrophoreses on 1.5% agarose gels as illustrated in Figure 2A. The three control samples of sugarcane were positive, and all three groups were positive which could be used for transcriptome sequencing.

### Functional Annotation of Unigenes

Dealing with the transcriptome sequencing of six tested sugarcane plants, 60,319,900 raw data were obtained in the experimental group and 70,729,262 in the control group. After removing the raw data with the linker and low-quality reads using Trimmomatic, clean data numbers that were obtained from the control and experimental groups were 65,845,402 and 55,528,852, respectively. Then clean data were processed to obtain transcript data and the assembled results are shown in Figure 2B. Following the screening criteria of *q*-value < 0.05 and | FoldChange| > 2, 1630 significant DEGs were obtained. Among them, the number of upregulated genes was 1,622 and the number of downregulated genes was eight. The biological replicates of the control group and the experimental group can meet the experimental requirements, as shown in Figure 2C. Significantly different DEGs in a biological functional annotation can divided into three major categories such as cellular components, molecular functions, and biochemical processes. Among these, the number of the biological functions of the cellular components was 22, and the molecular and biochemical levels were 20 and 26, as shown in Figure 2D. Thus, 68 biological functions can be divided into 11,847 small biological functions, of which 219 are related to viral protein synthesis and endoplasmic reticulum stress response, 77 are related to endocytosis, and 12 are related to PEX proteins. In the metabolic pathway analysis of DEGs, the designed pathways included cellular processes, environmental information processing, metabolism, and genetic information processing. Metabolic pathways were most enriched containing 12 pathways, while environmental information processing included only two pathways; membrane transport and signal transduction, as shown in Figure 2E. Further screening showed that there were 4 metabolic pathways (ko04144, ko04810, ko04530, and ko04520) related to the process of endocytosis, only ko04146 related to PEX proteins and seven metabolic pathways (ko03050, ko04140, ko04141, ko03010, ko03060, ko04068, and ko03008) were associated with the expression of viral protein synthesis and endoplasmic reticulum stress response.

### Differentially Expressed Genes in the Endocytosis Pathway

**FIGURE 3:**
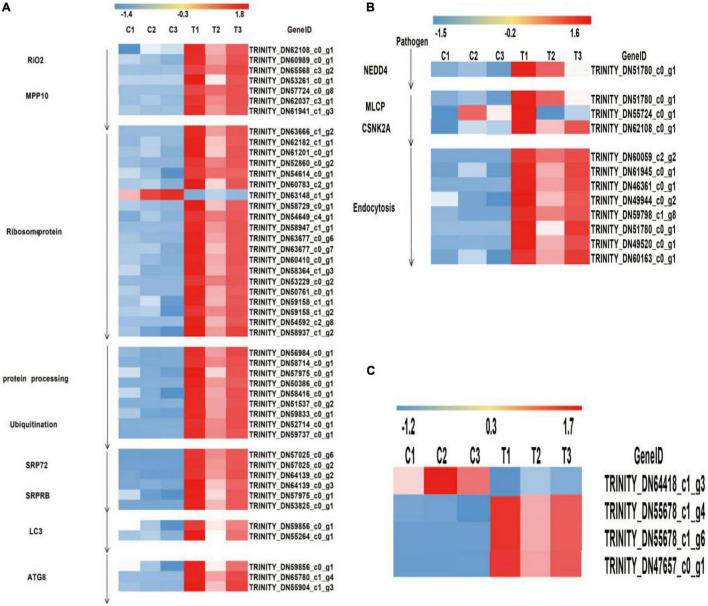
Heat map diagrams of relative expression levels of DEGs in response to plants under infection of ScYLV. **(A)** Heat map diagrams of relative expression levels of viral protein synthesis and endoplasmic reticulum stress response-related genes in plants. RIO2, RIO protein kinase right open reading frame 2; MPP10, M-phase phosphoprotein 10; SRP72, signal recognition particle 72; SRPRB, Recombinant Signal Recognition Particle Receptor B; LC3, microtubule-associated proteins light chain 3; ATG8, Autophagy-related protein 8. **(B)** Heatmap diagrams showed relative expression levels of twelve endocytosis genes in plants. NEDD4, E3 ubiquitin-protein ligase; MLCP, myosin light chain phosphatase; CSNK2A, casein kinase II subunit alpha. **(C)** Heat map diagrams of relative expression levels of PEX proteins. PEX5, peroxisomal biogenesis factor 5; PEX10, peroxisomal biogenesis factor 10; PEX13, peroxisomal biogenesis factor 13.

The aphids secreted the virus and saliva into the epidermal cells of the plant through the mouthparts, following virus transport to the vascular cells of the phloem of the plant through the intercellular filaments of the host, interacted with the clathrin receptor of the host cell and bound to the cell membrane. *NEDD4* activated the complex of *DLG2* and *DLG3*, which combined the complex with *TTAP1*, ZO-1, and JAM, increasing membrane fluidity and allowing the virus to transfer to the tight junction of the host cell. CSNK2A bound to actin inhibits by *MLCP* expression to promote actin rearrangement and inhibit actin contraction thus providing a channel for endocytosis. In this study, two enzymes were significantly expressed after viral infection, *NEDD4* (phosphorescent ubiquitin ligase) was controlled by one gene (TRINITY_DN51780_c0_g1), and *MLCP*-*CSNK2A* (myosin light chain phosphatase-casein kinase II subunit alpha) was co-expressed by three genes (the *MLCP* was co-regulated by TRINITY_DN51780_c0_g1 and TRINITY_DN55724_c0_g1, but *CSKN2A* was only regulated by TRINITY_DN-62108_c0_g1). Especially, TRINITY_DN51780_c0_g1 was also expressed in NEDD4 and endocytosis. Finally, the entire process of endocytosis had eight genes (TRINITY_DN60059_c2_g2-TRINITY_DN60163_ c0_g1), which were all upregulated under ScYLV infection. Based on the levels of gene expression, the DEGs heatmap was drawn using standard values, as shown in Figure 3B.

### Differentially Expressed Genes Involved in Viral Protein Synthesis and Endoplasmic Reticulum Stress Response Pathways

When the viruses entered the cells *via* endocytosis, the activity of the ER increased. The viral RNA entered the cytoplasm promoting the production of ribosomal RNA under the action of *RIO2* and *MPP10* and bound to it acting as a complex that translated the long peptide chain under the action of the ribosomal proteins. After translation, the newly synthesized long peptide chain was processed into protein and ubiquitinated to form a complex which could be recognized and cleaved by the protease, following the long peptide chain and was divided into structural and non-structural proteins under the action of *SRP72* and *SRPRB*. Due to the presence of many viral proteins in the cells, endoplasmic reticulum stress lead to increased expression of LC3 which then bound to E1 and E2 enzymes to form LC3-II -inducing autophagosome formation and ATG8 expression promoted autophagosomes in becoming a fused structure.

In the previous analysis, activity of ER was encoded by 22 proteins. To better understand the molecular mechanism of ER, DEGs were analyzed in this study. These proteins could be classified into nine families, among which the seven families were related to viral protein synthesis. *RIO2* (TRINITY_DN62108_c0_g1, TRINITY_DN60989_c0_g1, and TRINITY_DN65568_c3_g2) and *MPP10* (TRINITY_DN53261_ c0_g1, TRINITY_DN57724_c0_g8, TRINITY_DN62037_c3_g1, and TRINITY_DN61941_c1_g3) were co-regulated by seven genes. Ribosomal proteins were co-regulated by 20 genes, which included *Rpoc, B* (TRINITY_DN63666_c1_g2 and TRINITY_DN58729_c0_g1), *IF1* (TRINITY_DN62182_c1_g1, TRINITY_DN59158_c1_g1, and TRINITY_DN59158_ c1_g2), *SecY* (TRINITY_DN61201_c0_g1, TRINITY_DN54614_c0_g1, TRINITY_DN63148_c1_g1, TRINITY_DN54649_c4_g1, and TRINITY_DN54592_c2_g8), *L28e* (TRINITY_DN52860_c0_g2 and TRINITY_DN50761_c0_g1), *EF-TU, G* (TRINITY_ DN60783_c2_g1, TRINITY_DN58947_c1_g1, TRINITY_DN6 3677_c0_g6, and TRINITY_DN63677_c0_g7), L24e (TRINI TY_DN60410_c0_g1), *RpoA* (TRINITY_DN58364_c1_g3 and TRINITY_DN53229_c0_g2), and *S6e* (TRINITY_DN58 937_c1_g2), except TRINITY_DN63148_c1_g1 that is related to ribosomal structural protein *S17* and was downregulated while all the other genes were upregulated. Protein processing and ubiquitin protein were co-regulated by 9 genes, which included *Sec61* (TRINITY_DN57975_ c0_g1), *SAR1A* (TRINITY_DN58416_c0_g1), *Sec13/31* (TRINI TY_DN56984_c0_g1), and (TRINITY_DN58714_c0_g1), *OSTS* (TRINITY_DN50386_c0_g1), *ufd1* (TRINITY_D-N52714_c0_ g1), ufd2 (TRINITY_DN59737_c0_g1), *RAD23* (TRINITY_ DN59833_c0_g1), and *SEL1* (TRINITY_DN51537_c0_g2), while TRINITY_DN57975_c0_g1 also expressed in *SRPRB* and *SRP72, SRPRB*, and *SRP72* were regulated by six genes, among which *SRP72* (TRINITY_DN57025_c0_g6, TRINITY_DN57025_c0_g2, and TRINITY_DN64139_c0_g2) and *SRPRB*(TRINITY_DN64139_c0_g3, TRINITY_DN57975_ c0_g1, and TRINITY_DN53825_c0_g1) and were regulated by three genes each. When the process of viral protein synthesis finished, the endoplasmic reticulum stress response occurred, resulting in high expression levels of LC3 and ATG8 proteins. The expression of LC3 is regulated by the co-expression of two genes, and the expression of ATG8 is regulated by three genes. Especially, TRINITY_DN59856_c0_g1 was expressed in both ATG8 and LC3 expression. According to the ER gene expression levels, differentially expressed genes heatmap was drawn based on the standard values, as shown in Figure 3A.

### Differentially Expressed Genes in the PEX Proteins Pathway

Studies have shown that peroxisomes play an essential role in the infective host of psychopathologic fungi, especially the PEX gene involved in the formation and proliferation of peroxisomes, which has been identified in plant pathogens (Catto et al., 2022). In the RNA-Seq data, *PEX5* (TRINITY_DN47657_c0_g1) and *PEX13* (TRINITY_DN55678_c1_g4 and TRINITY_DN55678_c1_g6) were upregulated, but *PEX10* (TRINITY_DN64418_c1_g3) was downregulated. Means and variances of the six sets of data were calculated to get the standard value and construct the DEGs heatmap based on their standard values, as shown in Figure 3C.

### Validation of Differentially Expressed Genes by qRT-PCR

To verify the RNA-Seq data, qRT-PCR was conducted on 63 selected genes. According to RNA-Seq data, the selected genes were differentially expressed. For example, 19 endocytosis-related genes were upregulated except TRINITY_DN63148_c1_g1 which was downregulated. According to the literature, if the QRT-PCR result median value is more than 1, then the gene is upregulated. At the same time, transcription data are also upregulated, which showed that the results of QRT-PCR are consistent and reliable.

**FIGURE 4:**
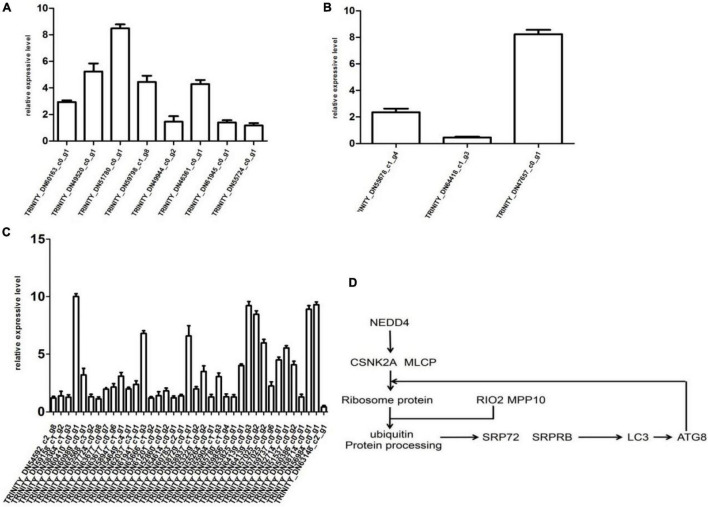
Differential expression multiple of the gene histogram shows genes express differentially in the control and test groups. Panels **(A–C)** represent endocytosis, PEX protein and viral protein synthesis, and ER stress response. Panel **(D)** represents the partial path of CP72-1210 response to ScYLV infection.

The results of qRT-PCR proved that the expression levels of 48 genes were consistent with transcriptome data, including endocytosis (TRINITY_DN60163_c0_g1, TRINITY_ DN49520_c0_g1, TRINITY_DN51780_c0_g1, TRINITY_DN59 798_c1_g8, TRINITY_DN49944_c0_g2, TRINITY_DN46361_ c0_g1, TRINITY_DN61945_c0_g1, and TRINI-TY_DN55724_ c0_g1), PEX protein (TRINITY_DN55678_c1_g4, TRINITY_ DN64418_c1_g3, and TRINITY_DN47657_c0_g1), and 37 genes associated with endoplasmic reticulum (TRINITY_DN54592_ c2_g8-TRINITY_DN6314-8_c2_g1). Briefly, the expression of TRINITY_DN60410_c0_g1 was increased by 10-fold as compared to control. Moreover, transcript levels of TRINI TY_DN64139_c0_g3, TRINITY_DN56984_c0_g1, and TRIN ITY_DN58714_c0_g1 were increased by 9-, 9-, and 8-fold, respectively, as compared to control. Relative expressive levels of genes in the CP72-1210 plants were calculated through qRT- PCR results, as showed in Figure 4.

## Discussion

Aphids, whiteflies, planthoppers, thrips, leaf beetles, and leafhoppers are the major vectors in the transmission of most of the viruses in plants (Catto et al., 2022). *M. sacchari* transmits the SCYLV in most of the tropical and subtropical regions in a persistent and circulative manner (Chinnaraja and Viswanathan, 2015). This role of aphids has already been confirmed in sugarcane through RT-PCR analysis by Chinnaraja and Viswanathan (Bertasello et al., 2021) and the effect of aphids on different genotypes of sugarcane by Bertasello et al. (2021). In this study, RT-PCR analysis (after 2 weeks of inoculation) of aphids, control, and CP72-1210 plants also confirmed the role of aphids in the transmission of SCYLV (Figure 2A). *De novo* sequencing of transcriptomes through RNA-Seq generated 2,31,870 transcripts with an N50 of 254 bp and an average length of 517 bp of CP72-1210 under SCYLV infection while 1,22,593 unigenes with average length of 455 and 579 bp N50 were generated (Figure 2B).

A total of 1,22,593 genes were assembled of which 1,630 genes were differentially expressed. Among DEGs, 1,622 were upregulated and eight were downregulated. Transcriptomic analysis can screen the genes related to aphid transmission virus, which has guiding significance for cutting off the transmission route by using RNAi of aphid virus transmission-related genes (Liang et al., 2021). Liang et al. (2021) analyzed the transcriptome of *M. persicae* with or without venomous peach aphid. The genes encoding epidermal proteins and the genes encoding ribosomal proteins play an important role in the process of aphids spreading plant viruses. In the omics study of aphid’s saliva secretion, transcriptome analysis and proteome analysis were performed, and after the two were combined, some proteins and expressed genes that may be related to virus transmission were found just like in the saliva of black-faced grasshopper (Tian et al., 2021) and whitefly (Grangeon et al., 2012).

Most the pathogenic viruses contain the core of RNA and with the help of endomembrane (ER, peroxisome, chloroplast, vacuole, etc.) form complex structures or inclusion bodies and replicate within the host cell (Agaoua et al., 2021). These membranes also take part in the transportation of viruses and their proteins (DeBlasio et al., 2015). In this research, the differentially expressed genes (DEGs) were functionally annotated into 68 biological functions, which were categorized into biochemical, molecular, and cellular components. The 68 biological functions were related to ER stress activity, viral protein synthesis, PEX proteins, and endocytosis.

Various proteins involved in clathrin-mediated endocytosis have been identified. These proteins also act in co-immunoprecipitation of PLRV (potato leafroll virus). In a previous study, PLRV was observed in the cytoplasmic vesicles (by transmission-electron microscopy) which tend to fuse with ER (near plasmodesmata), vacuoles, mitochondria, and nucleus and form vesicles (Alexander and Cilia, 2016). It was supposed that the function of these vesicles is to traffic the PLRV across the tissue barriers of aphids as well as plant hosts due to the presence of RTP (read-through protein) in viral capsids which aids in the transmission of the virus in both aphid vectors and plant hosts (Asadulghani et al., 2009). In this study, SCYLV interacted with the clathrin receptors bound with the cell membrane to form complexes/vesicles. Higher activity of *NEDD4* and *MLCP-CSKN2A* as well as their regulating genes were observed after viral infection which increased the membrane fluidity and allowed the transmission of the virus to the tight junction of cells. Furthermore, all the eight genes involved (as mentioned in Figure 3B) in the process of endocytosis were upregulated under infection of SCYLV.

In this study, the activity of the endoplasmic reticulum (ER) increased after the entry of the virus into the cell *via* endocytosis. Viral RNA under the combined action of *MPP10* and *RIO2* promotes the production of rRNA followed by translation and ubiquitination of viral RNA into structural and non-structural viral proteins under the combined action of *SRPRB* and *SRP72*. The presence of viral proteins triggers the ER stress response by increasing the expression of ATG8/LC3 and binding LC3 with E1 and E2 enzymes to form the LC3-II complex. This complex induces the formation of autophagosomes and ATG8 promotes the fusion of these autophagosomes to rid of viral bodies from the cell. Several studies had also shown the essential role of ATG8/LC3 in autophagy and its role in the antiviral defense response of plants which directly targets the degradation of virus or viral components (Gatica et al., 2018; Marshall and Vierstra, 2018; Johansen and Lamark, 2020; Ran et al., 2020; Wu et al., 2022). Recently, Johansen and Lamark (2020) observed that AT8/LC3 along with other autophagy proteins (ATG6/Beclint, ATG1, etc.) regulate the initiation, augmentation, and maturation of the autophagosomes. ATG8 targets the βC1 protein (a key factor for virus accumulation in plants) and degrades it, thus, protecting plants (Vietri et al., 2020). Similarly, ATG8 also directly targets the nucleoprotein C1 of TLCYnV and degrades it. XPO1 (exportin1) mediates the binding between C1 and ATG8 by transferring C1 in the cytoplasm from the nucleus (Ferreira et al., 2019). Once autophagosomes are formed, they are transported to lysosome/vacuole through the network of microtubules (Ferreira et al., 2021). In this research, the genes involved in the regulation of ATG8/LC3 were also studied and a heat map was drawn (Figure 3A).

Peroxisomes are the ubiquitous organelles having a significant role in the antiviral response metabolism of ROS and lipids and have an impact various important diseases in plants (Wang et al., 2019). The significance of peroxisomes in establishing the antiviral response of cells has been observed in various reports in the association of peroxisome-dependent signaling. The upregulation of *PEX5* and *PEX13* was observed under ScYLV infection and a heat map (Figure 3C) was drawn as well in this experiment. Similarly, the significance of *PEX13* and *PEX14* for the biosynthesis of peroxisomes, host infection, pathogenicity, and development of *Magnaporthe oryzae* was observed by Wang et al. (2019). In future experiments, data of this research will further explore the infection mechanism of yellow leaf disease and aid with future research.

## Conclusion

In this study, comparative transcriptome analysis was conducted between ScYLV-infected and ScYLV-free plants of CP72-1210. Data analysis of plants yielded 23,180 transcription data and obtained 1,22,593 unigenes by redundancy. GO functional enrichment and KEGG analysis of DEGs showed that they were mainly involved in endocytosis, PEX proteins synthesis, and ER stress response to viral protein synthesis, which included 364 GO functions and 12 KEGG pathways. Among them, there were 77 GO functions and four KEGG pathways related to endocytosis, relevant to 12 expressed genes and 219 GO functions and seven KEGG pathways were associated with an ER stress response to viral protein synthesis, corresponding to 47 expressed genes. Also, it had 12 GO functions and 1 KEGG pathway related to PEX proteins. The corresponding number of expressed genes was four. And we found 47 genes expression consistent with the transcription data based on the results of qRT-PCR including seven and three for endocytosis and PEX proteins, respectively. However, 37 genes were related to ER response function. Besides these, our research first discovered that ScYLV can induce the formation of autophagosomes in the analysis of ER stress. Therefore, this study proposed the first partial virus infection process, including virus entrance to the cell and viral protein synthesis leading to the foundation of the complete mechanism proposed for the future and offered an overall comprehension of sugarcane responses to ScYLV infection at the gene expression level.

## Data Availability Statement

We have deposited the raw data for 921674 “Transcriptome Analysis of Sugarcane Response to Sugarcane Yellow Leaf Virus Infection Transmitted by the Vector Melanaphis sacchari” in the Figshare platform. The associated download links are shown below: CPC three samples (https://figshare.com/articles/dataset/Sugarcane_RNA-seq_CPC/19802656). CPT three samples (https://figshare.com/articles/dataset/Sugarcane_RNA-seq_CPT/19808218).

## Author Contributions

All authors contributed significantly to conceptualization, experimentation, writing, review and editing of the current manuscript. All authors agreed to the submitted version of the current manuscript.

## Conflict of Interest

The authors declare that the research was conducted in the absence of any commercial or financial relationships that could be construed as a potential conflict of interest.

## Publisher’s Note

All claims expressed in this article are solely those of the authors and do not necessarily represent those of their affiliated organizations, or those of the publisher, the editors and the reviewers. Any product that may be evaluated in this article, or claim that may be made by its manufacturer, is not guaranteed or endorsed by the publisher.

## Footnotes

1
https://www.statistix.com

## Abbreviations

ScYLD: sugarcane yellow leaf disease
ScYLV: sugarcane yellow leaf virus
ScYLS: sugarcane yellow leaf symptom
BWYV: beet western yellows virus
BYDVPAV: barley yellow dwarf virus PAV
PLRV: potato leaf roll virus
AGO1: argonaute-1
ER: endoplasmic reticulum
DGE: digital gene expression
RPKM: reads per kb per million
DEGs: differentially expressed genes
RIO2: RIO protein kinase right open reading frame 2
MPP10: M-phase phosphoprotein 10
SRP72: signal recognition particle72
SRPRB: recombinant signal recognition particle receptor B
LC3: microtubule-associated proteins light chain 3
ATG8: autophagy-related protein 8
NEDD4: E3 ubiquitin-protein ligase
MLCP: myosin light chain phosphatase
CSNK2A: casein kinase II
PCR: polymerase chain reaction
PEX: peroxisome
CP: coat protein
QRT-PCR: quantitative real-time PCR
EST: expressed sequence tag
DLG2: disks large protein 2
DLG3: disks large protein 3
TJAP1: tight junction protein 4
zo-1: zonula occludens-1
JAM: junctional adhesion molecule 1
ufd1, ufd2: ubiquitin-recognition protein
EF-TU, G: elongation factor Tu elongation factor G
RPOC, B: RNA polymerase D’ subunit and β subunit
SecY: the central subunit of the protein translocation channel
L28e: large subunit ribosomal protein L28e
L24e: large subunit ribosomal protein L24e
RpoA: RNA polymerase α subunit
S6e: small subunit ribosomal protein S6e
S17: ribosomal structural protein S17
Sec61: protein transport protein Sec61 subunit alpha
SAR1: GTP-binding protein SAR1
Sec13/31: protein transport protein SEC13
OSTS: oligo saccharyl transferase complex subunit alpha
RAD23: UV excision repair protein RAD23
SEL1: SEL1 protein
TLCYnV: tomato leaf curl Yunnan virus.
